# Can Robotic Gait Training with End Effectors Improve Lower-Limb Functions in Patients Affected by Multiple Sclerosis? Results from a Retrospective Case–Control Study

**DOI:** 10.3390/jcm13061545

**Published:** 2024-03-07

**Authors:** Mirjam Bonanno, Maria Grazia Maggio, Laura Ciatto, Rosaria De Luca, Angelo Quartarone, Angela Alibrandi, Rocco Salvatore Calabrò

**Affiliations:** 1 IRCCS Centro Neurolesi Bonino Pulejo, 98124 Messina, Italy; mirjam.bonanno@irccsme.it (M.B.); laura.ciatto@irccsme.it (L.C.); rosaria.deluca@irccsme.it (R.D.L.); angelo.quartarone@irccsme.it (A.Q.); roccos.calabro@irccsme.it (R.S.C.); 2 Unit of Statistical and Mathematical Sciences, Department of Economics, University of Messina, 98122 Messina, Italy; aalibrandi@unime.it

**Keywords:** end effector, multiple sclerosis, neurorehabilitation, quality of life, robotic gait training

## Abstract

**Background:** Multiple sclerosis (MS) is characterized as a neurodegenerative condition possibly triggered by autoimmune mechanisms, impacting the entire central nervous system. In this context, neurorehabilitation plays a crucial role in every phase of the disease, aiming to restore and preserve motor functions in MS patients. In particular, robotic gait training (RGT) allows intensive, repetitive, and task-oriented training, which is pivotal in boosting neuroplastic processes. Thus, the primary aim of our study is to evaluate the effectiveness of innovative robotic gait training, using the G-EO system, on gait, functional abilities, and quality of life (QoL) in patients affected by MS. Secondly, we evaluated the effect of the robotic rehabilitation on lower-limb motor functioning, balance, sensation, and joint functioning. **Methods:** The study involved twenty MS patients, divided into two groups with comparable medical characteristics and rehabilitation training duration. The experimental group (EG) underwent robotic gait training with the G-EO system (n. 10), while the control group (CG) received traditional rehabilitation training (n. 10). **Results:** Both groups exhibited improvements in disability level (Functional Independence Measure), 10 m walking distance (10MWT), gait, and balance performance (Functional Ambulation Classification, Tinetti Scale). However, the EG demonstrated a more significant improvement. The G-EO system notably reduced spasticity in the lower limbs (Modified Ashworth Scale) exclusively in the EG. **Discussion:** This study suggests that the G-EO system could be a valuable tool for enhancing gait functions, including lower-limb movements, functional abilities, and QoL in individuals with MS.

## 1. Introduction

Multiple sclerosis (MS) is characterized as a neurodegenerative condition possibly triggered by autoimmune mechanisms, impacting the entire central nervous system [1,2]. In Italy, the estimated prevalence varies between 122 and 232 cases per 100,000 individuals in mainland regions and Sicily, averaging at 176 cases per 100,000 people [3]. Generally, the onset of MS occurs in early adulthood, negatively impacting functional outcomes and quality of life [4]. Symptoms are varied and heterogeneous since they depend on the localization of demyelination plaques throughout the brain [5]. Regarding mobility, patients with MS can experience fatigue, changes in gait due to ataxia, muscle weakness, spasticity, and sensory and proprioceptive deficits [6]. In addition, spasticity in the quadriceps, weakness of the hamstrings, and gastrocnemius tend to reduce hip extension during the stance phase of gait, as well as knee flexion during the swing phase [6,7]. These biomechanical alterations of gait are more pronounced when MS patients manifest spasticity. Even people with mild MS have shown gait abnormalities when they are compared to healthy people, as observed by various studies [8,9]. By or large, MS patients, compared to healthy controls, can often manifest alterations in the temporal parameters of gait, causing a decreased velocity, stride duration, and cadence. These gait alterations can increase the risk of falling and they are also associated with a worse quality of life [10]. Furthermore, MS patients present differences in gait alterations among the two main clinical subtypes, relapsing–remitting MS (RR-MS) and progressive MS (PR-MS). According to some authors [10,11], gait patterns seem to depend on the level of disability, suggesting that PR-MS may have worse gait parameters compared to RR-MS. The level of disability in patients with MS can be measured with the Expanded Disability Status Scale (EDSS), proposed by Kurtzke in 1983 [12]. This encompasses eight distinct systems, the pyramidal, cerebellar, visual, perceptual, cognitive, visceral, cerebral, and brainstem levels [12]. Additionally, it categorizes patients based on their residual walking capacity. Typically, scores up to 3.5 indicate mild-to-moderate impairment of functional autonomy, whereas scores surpassing 7.5 indicate severe disability, often resulting in wheelchair dependency or bed confinement [13]. In this context, neurorehabilitation plays a crucial role in every phase of the disease, aiming to restore and preserve motor functions in MS patients [14]. Conventional rehabilitation training includes physical exercise, which is a well-known approach to improving cardiorespiratory fitness, muscle strength, flexibility, and balance [15]. Physiotherapy treatments emphasizing gait training have consistently demonstrated their effectiveness in enhancing gait and mobility, while also mitigating the risk of falls [16]. However, when patients with MS experience increased difficulties in walking and decreased levels of independence, clinicians must adopt other rehabilitation strategies. In this sense, robotic gait training (RGT) allows intensive, repetitive, and task-oriented training, which is pivotal in boosting neuroplastic processes [17]. Among robotic tools, there is a distinction between exoskeletons and end effectors, based on their biomechanical features [18]. The first category of robots can be further distinguished as fixed or tethered exoskeletons like Lokomat (Hocoma, AG, Volketswil, Switzerland), and as portable or untethered exoskeletons for overground gait, like Ekso-GT and Indego (Ekso-Bionics, San Rafael, CA 94901, USA). Both types consist of wearable powered orthoses that match the joint of the device to the joint of the subject. Our previous study [19] demonstrated that Ekso (Ekso Bionics, USA) can be a valid tool to promote functional recovery in MS patients. Indeed, exoskeletons produce an automatized overground gait, with different degrees of assistance, improving motor outcomes, such as balance and gait. Differently from such exoskeletons, the end effectors are stationary devices with a single distal control requiring active participation. End effectors allow constant contact between feet and the moving platform, simulating gait phases [20]. Among end effectors for lower limbs, the G-EO system provides a propulsive motion of the legs thanks to two footplates, alternating stance and swing phases of gait, also simulating ascending and descending gait. Despite its potential, the role of the G-EO system in improving lower-limb functions in MS patients has not been yet investigated.

For these reasons, the primary aim of our study is to evaluate the effectiveness of an innovative RGT, using the G-EO system, on gait, functional abilities, and QoL in patients affected by MS. In addition, as a secondary aim, we evaluated the effect of robotic rehabilitation on lower-limb motor functioning, balance, sensation, and joint functioning only in the EG.

## 2. Materials and Methods

### 2.1. Study Design and Population

All patients included in the study were diagnosed with relapsing–remitting multiple sclerosis (RRMS). Twenty MS patients, who attended the Robotic and Behavioral Neurorehabilitation Unit of the IRCCS Centro Neurolesi “Bonino-Pulejo” between June 2018 and November 2019, underwent evaluation for inclusion in the analysis through an electronic recovery data system.

This retrospective case–control study adhered to the principles of the 1964 Helsinki Declaration and received approval from our Research Institute Ethics Committee (ID: IRCCSME 43/2018). The retrospective nature of the study and the extraction of data from electronic medical records helped minimize scoring bias. Motor and cognitive parameters were utilized to select suitable MS patients for inclusion in the analysis.

The MS patients included received rehabilitation with either the G-EO System or conventional approaches, based on their initial rehabilitation assignments. Retrospective evaluations, conducted at the onset and conclusion of training, were carried out by a multidisciplinary rehabilitation team comprising a neurologist, physiatrist, nurse, physiotherapist, and psychologist.

Inclusion criteria were as follows: (i) diagnosis of RRMS according to the revised McDonald criteria [21]; (ii) stable therapy for a minimum of six months before entry into the study; (iii) ability to walk independently (Functional Ambulation Classification—FAC ≥ 2); (iv) patients aged between 18 and 75 years.

Otherwise, patients were excluded if they presented the following criteria: (i) age > 75 years; (ii) diagnosis of concurrent psychiatric conditions or other significant medical comorbidities; (iii) presence of deficits (e.g., cognitive, visual, or auditory) that could limit the comprehension and/or execution of the proposed exercise; (iv) comorbidities that prevented upright posture and walking (e.g., hypotension); (v) refused consent or were unable to provide informed consent; (vi) recent bone fractures. In addition, we excluded patients if they had contraindications to the use of the technological instrumentation such as a weight > 150 kg and open lesions or bandages in contact with the harness.

### 2.2. Data Collection

Demographic and clinical information were retrospectively collected from all gathered patients. The outcomes obtained, along with details of the rehabilitation sessions, were documented. The data were collected retrospectively and subsequently analyzed. Prior to participation, patients provided general informed consent for the use of their data for research purposes.

### 2.3. Procedures

The included patients were divided into two groups, sharing similar medical characteristics and duration of rehabilitation training. However, they diverged in the type of rehabilitation approaches. Finally, the groups differed in demographic characteristics due to the small sample size.

The experimental group (EG) underwent robotic gait training (RGT) with the G-EO system (n. 10), while the control group (CG) received traditional rehabilitation training (n. 10).

Our rehabilitation protocol consisted of 40 training sessions each lasting around an hour for both groups (i.e., five sessions per week for eight weeks, in accordance with our established standard and clinical research protocols).

All patients underwent a clinical visit and neuropsychological evaluation at the beginning (T0) and the conclusion of the rehabilitation program (T1). In our neurorehabilitation unit, patients who undergo robotic rehabilitation treatments receive further clinical evaluations, to understand the effects of robotics on body segmental outcomes. This is why MS patients in the EG were also evaluated with the Fugl-Meyer–Lower Extremity assessment at T0 and T1.

### 2.4. Outcome Measures

Outcome measures were administered by a physiotherapist (LC) and a psychologist (MGM).

The primary outcomes, the motor and functional scales administered by the physiotherapist at baseline (T0) and post-treatment (T1), included the following: (i) The 10 Meters Walking Test (10MWT) [22] to assess walking speed in meters per second over a short distance. (ii) The Functional Ambulation Category (FAC) [23], a 6-point assessment tool used to evaluate the functional walking ability of patients. This considers the level of assistance required by patients during walking. (iii) The Tinetti scale (TS), made up of 16 items, with seven items assessing gait and nine items evaluating balance. A total score of 19 or less on the scale indicates a high risk of falls [24]. (iv) The Modified Ashworth Scale (MAS) [25] was used to assess spasticity in lower limbs. (v) The Functional Independence Measure (FIM) [26] was utilized to assess overall functioning across six subscales—self-care, sphincter control, transfer, locomotion, communication, and social cognition ability. A higher score indicates less disability for basic daily functions. On the other hand, the psychological measure administered by the psychologist consisted of the Quality of Life-54 Multiple Sclerosis (MSQoL-54) questionnaire [27] to evaluate the quality of life related to physical and emotional aspects in individuals suffering from MS.

Finally, for the secondary outcome, EG patients were also evaluated with the Fugl-Meyer–Lower Extremity (FMA-LE) assessment [28], which assesses motor functioning at the hip, knee, and ankle, coordination, reflexes, sensory functioning, balance, joint range of motion, and joint pain.

### 2.5. G-EO System

The G-EO System (Reha Technology, Olten, Switzerland) [29] is a robotic end effector that features footplates (pedals) for lower-limb movement. These footplates facilitate movement from the bottom to the top with fully programmable trajectories. Parameters such as step length (up to 550 mm), step height (up to 400 mm), footplate angles (up to 90 degrees), velocity of movements (up to 2.3 km/h), and acceleration peak (up to 10 m/s^2^) can all be adjusted according to specific requirements. The actuation of leg movement does not come from hips and knee, but from ankles. In particular, the patients’ feet are stitched into the footplate’s thorough straps. In this way, the device simulates walking, performing forward and backward movements, and ascending and descending stairs. Additionally, the device is outfitted with handrails on both sides and incorporates a body weight support (BWS) system.

**Figure 1:**
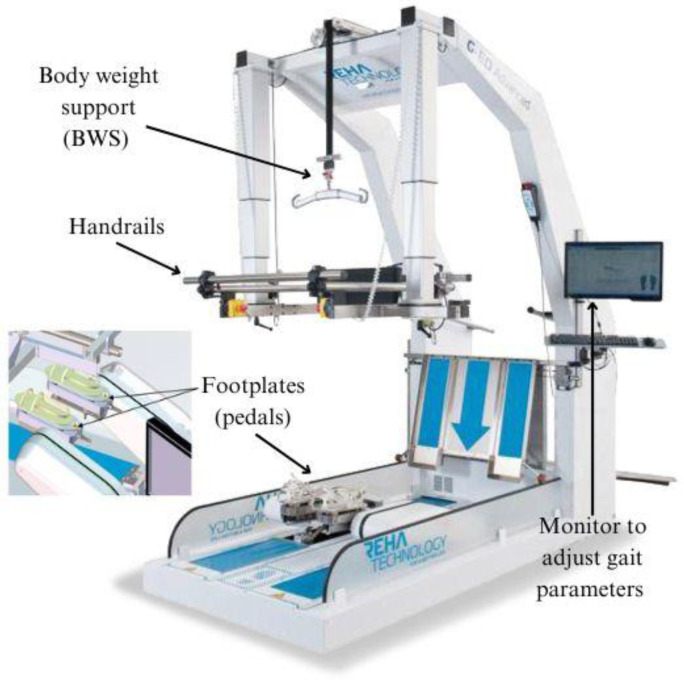
The G-EO system and its components.

This BWS system guarantees the vertical displacement of the patient’s center of mass (CoM), which is controlled by a precise regulation of the patient’s lateral movement. For a visual elucidation, consult Figure 1.

During the sessions, the patients initiated walking at a comfortable pace of approximately 0.4 m/s, progressively increasing by 0.5 m/s every three minutes until reaching the maximum tolerated velocity. Once the patient achieved the maximum tolerated velocity, the session commenced. This technique was repeated daily. If patients were able to maintain step length, cadence, step number, and stride length safely, the velocity was maintained; otherwise, training continued at the velocity of the previous session. Step cadence remained constant in each session. The intensity of training progressed individually to prevent fatigue, with patients closely monitored. In cases of fatigue, walking speed was reduced to a comfortable pace (approximately 25% lower).

Similarly, the time required for ascending/descending stairs (step by step in an alternating pattern) and the pace (stairs/min) were determined. Both parameters were adjusted similarly to floor walking, with the step rise standardized at 18 cm and the vertical (lateral) hip displacement at 5 ± 2.5 cm.

**Figure 2:**
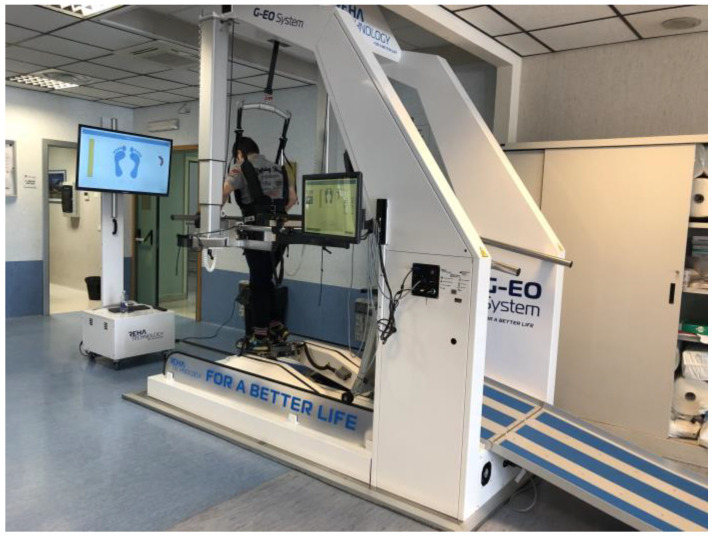
MS patient performing gait training on G-EO system at Robotic and Behavioral Neurorehabilitation Unit of the IRCCS Centro Neurolesi “Bonino-Pulejo”.

Initially, body weight support (BWS) was set at 80% discharge for both floor walking and stair climbing, gradually decreasing by 10% each week until reaching 10% or the highest tolerated BWS, which was compatible with the patient’s tolerance and fatigue levels. Refer to Figure 2 for a visual representation.

#### Conventional Gait Training

Patients in the control group (CG) underwent conventional gait training (CGT), which included various exercises such as weight-shifting, core muscle strengthening, monopodal and bipodal balance exercises, and gait training involving obstacles, tandem, and slalom walking. Like the experimental group (EG), the intensity of training in the CG was individually tailored to prevent fatigue, with close monitoring of patients. Additionally, throughout all sessions, MS patients received manual guidance and supervision from physiotherapists to minimize the risk of falls.

### 2.6. Statistical Analysis

The data were analyzed using GraphPad Prism version 7.0, with statistical significance set at *p* < 0.05. Descriptive statistics were presented as mean (standard deviation) or median (first–third quartile) for continuous variables, while frequencies (%) were used for categorical variables. Given the small sample, we decided to use a non-parametric approach. Thus, we used the Wilcoxon test for paired data comparisons, particularly in evaluating patient outcomes at different time points. This test was implemented as two-tailed, where appropriate. Additionally, we used the Mann–Whitney U test to compare groups based on some demographic factors, such as education, and baseline test scores. We used Fisher’s exact test to compare the two groups in terms of sex and medication.

Finally, to identify predictors of treatment response, linear regression models were estimated for all clinical scales at T1, entering age, sex, EDSS, and time since onset as covariates. Normality of distribution of dependent variable was tested using the Shapiro–Wilk test, the homoscedasticity of the data was examined using Bartlett’s test, and the presence of multicollinearity among the explanatory variables was evaluated by means of Variance Inflation Factor (VIF). Once the normality in distribution of dependent variables, the homoscedasticity, and the absence of multicollinearity had been checked, we estimated the regression models on the whole sample of examined subjects. When appropriate, correlations between variables were calculated using the Spearman coefficient. Analyses were performed using a GraphPad software package (version 10.2.0). A confidence level of 95% was set with an alpha error of 5%. Statistical significance was set at *p* < 0.05.

## 3. Results

**Figure 3:**
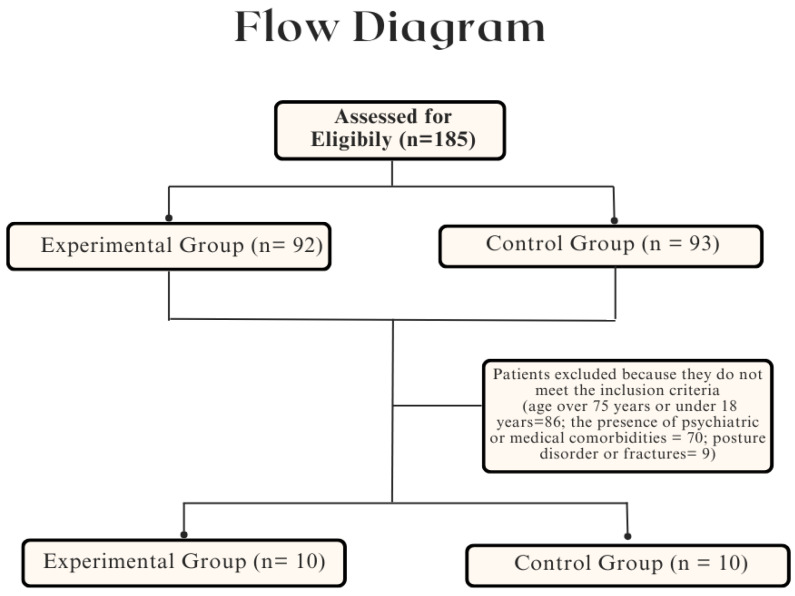
Flow diagram of patient selection process.

The medical records of 185 MS patients treated in our unit were examined. We assessed and excluded 165 patients based on the inclusion criteria (Figure 3). MS patients were excluded because they were older than 75 years or younger than 18 years (86 patients); or because they had psychiatric or medical comorbidities (70 patients); or had previous fractures (9 patients). The final sample comprised 20 MS patients, divided equally between the EG (n. 10) and the CG (n. 10).

All patients completed the study without any significant side effects and both groups received the same amount of rehabilitation training. No significant differences were observed in age (*p* = 0.24), education (*p* = 0.98), sex (*p* = 0.08), medical characteristics (disease duration (*p* = 0.68), or EDSS score (*p* = 0.41) between the EG and CG. The final sample consisted of 20 MS patients (10 females and 10 males, with a mean age of 52.5 (10.4)). A more detailed description of the sample is reported in Table 1. No significant differences were found between the two groups at T0 except for the MSQoL Mental and Physical scores (see Table 2).

Regarding the primary outcome, the results of the Wilcoxon’s tests showed that both types of rehabilitation led to an improvement in the level of disability (FIM), an increase in the distance covered in 10 m (10MWT), and in the performance in gait and balance (TS), although the statistical significance was greater (FIM, *p* < 0.001; 10MWT, *p* < 0.001; TS, *p* < 0.002) in the EG. Additionally, the G-EO System had an impact on reducing spasticity in the lower limbs (MAS) exclusively in the EG. Both groups also demonstrated an enhancement in the perception of quality of life (QoL), with a more significant improvement in physical and mental perception observed in the EG (Table 3).

For all clinical scales at T1, entering age, sex, EDSS, and time since onset as covariates, we observed the following (Table 4): Mental MSQoL is significantly influenced by sex, particularly in males, associated with higher levels of Mental MSQoL. The outcome 10 MTW at T1 is influenced by time since the onset of disease. Specifically, the negative coefficient indicates that lower duration of disease onset is associated with higher levels of the outcome 10 MTW at T1. FAC is influenced by age, whereby younger subjects exhibit significantly higher levels of FAC and tend to score higher on the Tinetti test as well.

Concerning the secondary outcome, we observed improvement in almost all motor functions, including those related to hip, knee, and ankle, coordination, reflexes, and balance, as indicated by the FMA-LE subscales (Table 5).

## 4. Discussion

As far as we know, this is the first study investigating the outcomes of patients with MS trained with the G-EO System device. Our results revealed that this innovative training improved both motors (FMA-LE, TS,10MWT), functional outcomes (FAC, FIM), spasticity (MAS), and quality of life (MSQoL). In particular, we have registered improvements in lower-limb movements (FMA-LE), gait function (TS, FAC), spasticity (MAS), and gait acceleration (10MWT). Our findings are likely influenced by the suitability of end-effector devices for patients who had residual locomotor function, indicating a sufficient activation of proximal joints and muscles [30]. Most MS patients manifest a hemiparetic gait due to spasticity and stiffness localized at the knee and ankle [6,31]. This could lead to reduced ankle dorsiflexion during initial contact and insufficient plantarflexion during the pre-swing phase of the gait [31]. Moreover, the use of end effectors for gait training could improve the insufficient inter-limb ankle–knee–hip coordination of MS patients during gait [32]. The end-effector robot functions by securing the patient’s feet onto separate footplates, which move along programmed gait trajectories for both the vertical and horizontal components of the center of mass (COM). It offers guidance and real-time visual feedback to the patient during the process [33]. During training with the G-EO system, the gait cycle became as nearly physiological as possible, with a reduction in pathological co-activation. This system facilitates ankle dorsiflexion and plantar flexion more than a grounded exoskeleton, like the Lokomat, can. In fact, exoskeletons applied forces at hip and knee plus passive guidance provided by robotic motors [34]. Other commercially available exoskeletons, such as the Ekso-GT and Indego, have also been investigated in the context of gait training in patients with MS, as well as the Lokomat. However, it is difficult to compare our results with these findings because of the substantial differences in device functionality. For example, Ekso-GT is an overground exoskeleton, providing motorized assistance at hip and knee, while the GE-O system only provides support on the foot. As already demonstrated by some authors [35,36], Ekso-GT is a feasible and effective device to improve gait speed and independence during walking. Otherwise, Keeogo [37] provides motorized assistance only at the knees. The main difference between Keeogo and other exoskeletons is that it is lighter than Ekso-GT and it can also be used at home and not only in clinical settings. However, we did not find studies comparing end effectors with exoskeletons. In this vein, future studies could investigate which robotic device, between the exoskeleton and the end effector, is better to improve gait abilities in MS patients [38].

Moreover, as demonstrated in our previous work, the G-EO system seemed to influence cortical excitability in patients affected by spinal cord injury [39]. Intensive, repetitive, assisted-as-needed, and task-oriented training can enhance motor learning by engaging both the efferent motor pathways and afferent sensory pathways throughout the training process [40]. This dual activation does have a role not only in enhancing cortical excitability but also in activating the central pattern generator (CPG) in the spinal cord. What is more, CPG receives and elaborates sensorimotor information coming from supraspinal centers (corticospinal drive and extra-pyramidal descending output) and peripheral inputs [41]. In fact, walking with end effectors allows high degrees of freedom motions, which may provide a more realistic gait [42]. This aspect could have promoted an effective recovery instead of relying solely on behavioral compensation mechanisms.

Thus, repetitive movements can serve as a foundational element in the acquisition of motor skills, promoting muscle memory and improving motor coordination. Repetition allows patients to hone specific movements, promoting greater efficiency and precision over time [16]. This approach is particularly useful in activities that require precise and consistent motor control, such as gait training or fine motor skill development. Furthermore, the incorporation of strategies during training, such as the presence of obstacles and the diversification of paths, introduces elements of complexity and adaptability to therapeutic sessions [16,35]. These strategies mimic real-world challenges, encouraging patients to engage in problem solving and adapt their movements accordingly. By incorporating different stimuli and environmental conditions, therapy sessions become more dynamic and reflect the challenges encountered in everyday life. Finally, repetitive movements lay the foundation for skill acquisition and motor refinement, and the integration of strategies adds depth and versatility to therapeutic sessions, promoting greater transferability of skills to real-world contexts [34]. A balanced approach that combines repetitive movements and strategic interventions may offer the most comprehensive and effective means of achieving therapeutic results.

An early rehabilitation intervention aimed at strengthening the residual components is supported by the results emerging from our linear regression. Indeed, our study shows that the duration of the disease influences the outcome of 10 MTW. This suggests that disease progression over time may impact motor skills as measured by this outcome. A shorter duration from onset could indicate an early stage of the disease, while a longer duration could indicate a more advanced stage. This aspect indicates the importance of an early rehabilitation intervention aimed at maintaining motor functioning over time. Furthermore, another aspect that influences the motor component is age, which influences FAC scores, with higher scores in younger subjects. This finding suggests a correlation between age and motor skills. This may be due to greater physical resilience and ability to maintain motor function in younger individuals.

The substantial involvement of the lower-limb distal muscle may possibly be explained by the engagement of the CPG through both ascending and descending inputs, which runs counter to what is typically seen after exoskeleton-based gait training [30]. However, certain characteristics are necessary for CPG activation, including a heel strike, which is impossible to replicate in end effectors. In this view, CPGs are not only activated through sensory and afferent inputs like the heel strike, but they also receive information from supraspinal areas. These inputs help initiate locomotion, activating CPGs, and adapt the pattern based on environmental cues [43]. This could rely on the end effector’s particular mode of operation, whereby the footplates drive bottom-to-top movements as opposed to the top-to-bottom movements provided by motorized orthoses. The dynamics of end effectors ensure that the footplates move along a natural trajectory throughout the phases of gait and closely mimic the natural movements of walking, which can help to stimulate the CPG [44].

Notably, the spasticity outcome (as per MAS) was significantly improved only in the EG, revealing that RGT could be superior to conventional gait training in reducing spasticity, as reported by other authors. It has been hypothesized that RGT, through the activation of neuroplastic processes, could contribute to regulating the corticospinal excitability mechanisms responsible for spasticity [45,46]. Spasticity refers to an increase in tonic stretch reflexes that is dependent on the velocity of movement, often accompanied by exaggerated tendon jerks [47]. In this view, RGT with its repetitive movements may reduce spasticity by activating spinal locomotor centers and modulating the corticospinal pathway.

In addition, our results revealed improvements in the balance subitem of FMA-LE, although our MS sample performed gait training. The improvements in balance observed through the G-EO system may be attributed to the concept of “reverse transfer”. This notion suggests that repetitive and highly intensive gait training could enhance non-walking tasks, including balance and postural stability [48,49].

Lastly, we found an improvement in perceived quality of life (MSQoL) in both groups at the end of the training. According to other authors [50,51], such a result can be interpreted as a positive impact of rehabilitation on QoL. In particular, the MSQoL evaluates both emotional and physical aspects of QoL, suggesting that specific and effective treatment may have a positive impact on how patients perceive QoL. In this vein, the necessity of tailoring rehabilitation treatment according to patients’ needs becomes clear. Indeed, comprehending the impact of robotic therapies on the quality of life of people with MS is essential for evaluating the effectiveness of such interventions [19]. While the improvement of mobility and physical function through these therapies is often emphasized, it is equally crucial to examine their impact on patients’ overall well-being, considering physical, emotional, social, and psychological aspects. As highlighted in our studies, exoskeleton therapies can influence daily activities, social participation, emotional well-being, and health perception. Understanding patients’ perspectives on such therapies is crucial for tailoring interventions to their specific needs and preferences. Patients’ opinions provide valuable insights into the practical challenges, benefits, and limitations of exoskeleton therapies, thus guiding healthcare professionals in refining treatment protocols and optimizing the overall patient experience [52]. Patients’ feedback and high scores about their perceived motor and mental well-being highlights the necessity of incorporating the patient’s viewpoint within rehabilitative interventions. Additionally, previous studies have shown that patients find robotic rehabilitation attractive, adopting an active attitude without feeling stressed [53]. Moreover, high usability scores in healthy subjects and stroke patients undergoing robot-assisted therapy for upper-limb rehabilitation suggest the potential benefits of such approaches in managing neurological conditions [54]. It is interesting to note that sex appears to significantly influence the Mental MSQoL score, with higher scores observed in males. This might suggest a difference in the perception of health-related quality of life between men and women within the sample. Future study with a larger sample is needed to understand this important issue.

This retrospective study has several limitations worth acknowledging, including its retrospective design. The sample size is small, potentially limiting the generalizability of the results to the wider MS population. Furthermore, the absence of gait analysis, as well as kinematic and kinetic parameters, could have impacted the findings. Moreover, the two groups exhibited significant differences at baseline in terms of sex and quality of life. Consequently, it is challenging to accurately estimate the treatment effect. However, it is important to note that this study serves as an exploratory endeavor, underscoring the need for future clinical studies with larger sample sizes and more targeted motor and cognitive outcome measures to validate and expand upon our initial findings.

## 5. Conclusions

In conclusion, our findings suggests that end effectors, like the G-EO system, could be considered as a useful device to improve gait functions, including lower-limb movements, functional abilities, and QoL, in patients with MS. However, larger-sample randomized studies are needed to confirm these promising results and to investigate whether and to what extent they last over time.

## Figures and Tables

**Table 1:** Demographic and clinical characteristics of the patients.

	Experimental	Control	All	*p*-Value
Patients	10 (50.0%)	10 (50.0%)	20 (100%)	
Age	49.0 (10.0)	53.4 (10.7)	52.5 (10.4)	0.24
Education	11.3 (3.3)	11.6 (0.8)	11.9 (4.9)	0.98
Sex				
Male	8 (80.0%)	2 (20.0%)	10 (50.0%)	0.007
Female	2 (20.0%)	8 (80.0%)	10 (50.0%)	
Disease duration, (years)	10.7 (5.3)	10.1 (6.1)	10.5 (5.8)	0.68
Median EDSS	4.7 (1.4)	4.9 (0.4)	4.8 (1.0)	0.41
Therapy				
Avonex	3 (30.0%)	2 (20.0%)	5 (25.0%)	0.85
Tecfidera	2 (20.0%)	3 (30.0%)	5 (25.0%)	
Natalizumab	3 (30.0%)	3 (30.0%)	6 (30.0%)	
Alemtuzumab	2 (20.0%)	0 (0.00)	2 (10.0%)	
Fingolimod	0 (0.00)	2 (20.0%)	2 (10.0%)	

Mean (standard deviation) was used to describe continuous variables; proportions (numbers and percentages) were used to describe categorical variables.

**Table 2:** Statistical comparisons between the experimental (EG) and control (CG) groups at baseline (T0).

Clinical Assessment	Experimental Group	Control Group	*p*-Value *
	T0	T0	
10MWT	9.5 (7.4–11.3)	13.2 (7.5–22.8)	0.61
FAC	1.0 (1.0–4.0)	3.0 (1.7–3.2)	0.70
TS	15.5 (14.5–19.5)	16.0 (11.0–20.2)	0.70
MAS	0.7 (0.0–1.0)	0.3 (0.1–0.7)	0.30
FIM	105 (97–110)	115 (109.75–117.5)	0.09
MSQoL Ph	62.7 (54.7–78.5)	49.0 (41.6–64.8)	**0.05**
MSQoL MT	62.7 (54.7–78.5)	49.0 (41.6–64.8)	**<0.001**

* Statistical significances are in bold. The results are expressed as median (first and third quartiles). Legend: 10MWT: 10 Meters Walking Test, FAC: Functional Ambulation Classification, TS: Tinetti Scale, MAS: Modified Ashworth Scale, FIM: Functional Independence Measure, MSQoL Ph: Multiple Sclerosis Quality of Life of Physical Health Composite Score, and MSQoL MT: Multiple Sclerosis Quality of Life of Mental Health Composite Score.

**Table 3:** Statistical comparisons of clinical scores between baseline (T0) and follow-up (T1) were conducted for both the experimental (EG) and control (CG) groups.

Clinical Assessment	Experimental Group		*p*-Value *	Control Group		*p*-Value *
	T0	T1		T0	T1	
10MWT	9.5 (7.4–11.3)	9.6 (6.2–11.9)	**<0.001**	13.2 (7.5–22.8)	10.4 (5.1–28.1)	**0.002**
FAC	1.0 (1.0–4.0)	3.0 (2.0–4.0)	0.01	3.0 (1.7–3.2)	3.0 (2.0–4.0)	0.76
TS	15.5 (14.5–19.5)	18.0 (15.8–24.0)	**<0.001**	16.0 (11.0–20.2)	23.0 (15.0–26.0)	**<0.001**
MAS	0.7 (0.0–1.0)	0.1 (0–0.5)	**<0.001**	0.3 (0.1–0.7)	0.3 (0.0–0.6)	0.30
FIM	105 (97–110)	111.5 (107–116)	**<0.001**	115 (109.75–117.5)	121 (120–123.75)	**<0.001**
MSQoL Ph	62.7 (54.7–78.5)	86.7 (69.1–94.6)	**<0.001**	49.0 (41.6–64.8)	74.6 (68.7–83.3)	**<0.001**
MSQoL MT	62.7 (54.7–78.5)	86.7 (69.1–94.6)	**<0.001**	49.0 (41.6–64.8)	74.6 (68.7–83.4)	**<0.001**

* Statistical significances are in bold. The results are expressed as median (first and third quartiles). Legend: 10MWT: 10 Meters Walking Test, FAC: Functional Ambulation Classification, TS: Tinetti Scale, MAS: Modified Ashworth Scale, FIM: Functional Independence Measure, MSQoL Ph: Multiple Sclerosis Quality of Life of Physical Health Composite Score, and MSQoL MT: Multiple Sclerosis Quality of Life of Mental Health Composite Score.

**Table 4:** Linear regression models for treatment response outcomes.

Independent Variables		Unstandardized Coefficients		Standardized Coefficients	t	*p*-Value	95% CI for B	
		B	SD	Beta			Lower Limit	Upper Limits
		**Outcome: Physical MSQoL**						
	Sex	−18.75	10.76	−0.46	−1.74	0.10	−41.69	4.18
	Age	−0.45	0.45	−0.22	−1.00	0.33	−1.41	0.51
	EDSS	2.98	5.93	0.12	0.50	0.62	−9.65	15.63
	Time since onset	**0.01**	1.58	**0.002**	**0.01**	0.99	−3.36	3.38
		**Outcome: Mental MSQoL**						
	**Sex**	−40.00	18.14	−0.57	−2.20	**0.04**	−78.66	−1.33
	Age	−0.30	0.76	−0.08	−0.39	0.70	−1.92	1.32
	EDSS	7.86	10.00	0.20	0.78	0.44	−13.44	29.18
	Time since onset	−0.46	2.66	**−0.03**	−0.17	0.86	−6.15	5.22
		**Outcome: FIM t1**						
y	**Sex**	15.62	19.73.8	0.22	0.79	0.44	−26.44	57.70
	Age	0.81	0.83	0.24	0.97	0.34	−0.96	2.58
	EDSS	−1.98	10.88	−0.05	−182	0.85	−25.17	21.21
	Time since onset	−0.35	2.90	**−0.03**	−0.12	0.90	−6.54	5.84
		**Outcome: 10 MTW t1**						
	**Sex**	7.44	6.44	0.29	1.15	0.26	−6.28	21.17
	Age	−0.23	0.27	−0.18	−0.84	0.41	−0.80	0.34
	EDSS	−4.50	3.55	−0.30	−1.27	0.22	−12.07	3.06
	Time since onset	−2.00	0.94	−0.45	−2.11	**0.05**	−4.02	**0.02**
		**Outcome: FAC t1**						
	**Sex**	0.35	0.49	0.18	0.73	0.48	−0.69	1.41
	Age	−0.05	**0.01**	0.53	−2.78	**0.01**	−0.09	−0.01
	EDSS	0.54	0.29	0.46	1.85	0.09	−0.09	1.18
	Time since onset	0.09	0.06	0.27	1.42	0.18	−0.05	0.22
		**Outcome: Tinetti t1**						
	**Sex**	1.28	2.67	0.12	0.48	0.64	−4.42	6.99
	Age	−0.22	0.11	−0.44	−2.02	**0.06**	−0.46	0.01
	EDSS	0.56	1.47	0.09	0.38	0.70	−2.58	3.70
	Time since onset	0.72	0.39	0.40	1.84	0.08	−0.11	1.56

**Table 5:** Wilcoxon’s test of Fugl-Meyer–Lower Extremity (FMA-LE) assessment in the EG.

Clinical Assessment	Experimental Group		*p*-Value*
	T0	T1	
Motor functioning lower extremity	14.00 (10–17.7)	15.5 (12.5–19.5)	**0.002**
Balance	3.0 (2.2–3.7)	4.0 (4.0–4.0)	**<0.001**
Sensory functioning	12.0 (10.0–12.0)	12.0 (11.0–12.0)	0.10
Joint range of motion	20.0 (16.5–20.0)	20.0 (17.7–20.0)	0.52
Joint pain	20.0 (20.0–20–0)	20.0 (20.0–20–0)	0.34
Total	17.5 (12.2–21.7)	19.5 (16.5–24.5)	**<0.001**

* Statistical significances are in bold. The results are expressed as median (first and third quartiles). Legend: 10MWT: 10 Meters Walking Test, FAC: Functional Ambulation Classification, TS: Tinetti Scale, MAS: Modified Ashworth Scale, FIM: Functional Independence Measure, MSQoL Ph: Multiple Sclerosis Quality of Life of Physical Health Composite Score, and MSQoL MT: Multiple Sclerosis Quality of Life of Mental Health Composite Score.

## Data Availability

Data will be available on request to the corresponding author due to privacy reasons.
